# Early Resumption of Sex following Voluntary Medical Male Circumcision amongst School-Going Males

**DOI:** 10.1371/journal.pone.0168091

**Published:** 2016-12-08

**Authors:** Gavin George, Kaymarlin Govender, Sean Beckett, Carl Montague, Janet Frohlich

**Affiliations:** 1 Health Economics and HIV and AIDS Research Division, University of KwaZulu-Natal, Durban, South Africa; 2 Centre for the AIDS Programme of Research in South Africa, University of KwaZulu-Natal, Durban, South Africa; London School of Hygiene and Tropical Medicine, UNITED KINGDOM

## Abstract

Voluntary medical male circumcision is an integral part of the South African government’s response to the HIV and AIDS epidemic. Following circumcision, it is recommended that patients abstain from sexual activity for six weeks, as sex may increase the risk of female-to-male HIV transmission and prolong the healing period. This paper investigates the resumption of sexual activity during the healing period among a cohort of school-going males in the KwaZulu-Natal province of South Africa. The analysis for this paper compares two groups of sexually active school-going males: the first group reported having sex during the healing period (n = 40) and the second group (n = 98) reported no sex during the healing period (mean age: 17.7, SD: 1.7).The results show that 29% (n = 40) of young males (mean age: 17.9, SD: 1.8) who were previously sexually active, resumed sexual activity during the healing period, had on average two partners and used condoms inconsistently. In addition, those males that engage in sexual activity during the healing period were less inclined to practice safe sex in the future (AOR = 0.055, p = 0.002) than the group of males who reported no sex during the healing period. These findings suggest that a significant proportion of young males may currently and in the future, subject themselves to high levels of risk for contracting HIV post circumcision. Education, as part of a VMMC campaign, must emphasize the high risk of HIV transmission for both the males their partners during the healing period.

## Introduction

Voluntary medical male circumcision (VMMC) has become an integral part of the South African government’s response to the HIV and AIDS epidemic in South Africa, as highlighted in the National Strategic Plan [1]. VMMC is a key prevention tool within the strategy with ambitious targets set out in the national plan. Although VMMC has the potential to be an effective medical intervention for preventing HIV [2–4], there are some potential health risks once males have been circumcised. One concern is a heightened vulnerability to HIV transmission if engaging in risky sexual behaviour during the six week healing period [5].

VMMC in southern Africa is focused on engaging males in areas with high HIV prevalence and low circumcision rates, as recommended in the joint strategic action framework released by the WHO/UNAIDS [6]. Scaling up of VMMC amongst young males before they enter a period of high risk, due to increased sexual activity, has been demonstrated to be an effective and efficient HIV prevention strategy in South Africa [7].

However, the possibility of risk compensation following VMMC is a concern [8]. That is, VMMC may increase risky sexual behaviour among males who have undergone circumcision [8–11]. Following circumcision it is possible that the perceptions of the risk of contracting HIV may diminish with a corresponding increase in risky sexual behaviour [8]. The net effect is higher levels of susceptibility to HIV and STI infection. To date, evidence has shown that VMMC is not associated with risk compensation and generally young males who have been circumcised have decreased their sexual risk [12, 13].

Of further concern to policymakers is the early resumption of sexual activity during the healing period, as this phenomena may have a negative impact on the public health benefit conferred to males following the removal of the foreskin. Approximately 75% of patients’ VMMC-related wounds heal within four weeks of the procedure [2, 14]. This, increases to 93% at six weeks after the operation. Thus following circumcision, it is recommended that males abstain from sexual activity for the six weeks following the procedure. Sexual activity during this period may prolong the healing process, and in high HIV prevalence settings, increase the probability of HIV acquisition amongst those recently circumcised. While the findings from a study that analysed three randomised control trials—which assessed the efficacy of VMMC as a preventative measure of HIV/AIDS—concludes that early resumption of sex during the healing period is not associated with HIV risk; the authors have cautioned that the study power is limited and the results should not be considered definitive [14]. The authors do however conclude that sex should be avoided until after the healing period.

Modelling work based on the early resumption of sex following medical circumcision in Zambia, indicates that should the percentage of men resuming sex during the healing period reach a certain threshold, there would be an increase in the number of HIV infections amongst women in the first year of the VMMC programme [5]. The study determined that the effect of VMMC on reducing HIV acquisition amongst women is dependent on the prevalence of sex during the healing period and whether only HIV negative men were circumcised. The model suggested that should 30% or more of circumcised men resume sex during the healing period and the HIV prevalence of these males is 6%, the programme would have generated more new infections among women in the year following circumcision than it would have averted. The Zambian study concludes that the net benefit of VMMC for HIV prevention amongst men and women will not be offset by resumption of sex during the healing period as more HIV infections will be averted as coverage of VMMC increases [5]. However, to ensure the greatest possible number of HIV infections are averted, sex during the healing period should be avoided.

Educational messages to discourage sexual activity during the six week healing period have lacked visibility in VMMC campaigns, in comparison to other messages such as double protection through condom use. This may be partly due to a dearth of research on gauging the prevalence of sexual activity during this critical period and a lack of analysis on the factors associated with the early resumption of sex. In addition, available studies have focused on men 18 years and older. In this regard, findings from studies in Kenya and Zambia highlight that the strongest predictor of engaging in sexual activity during the healing period is being married or cohabiting and having a history of engaging in risky sexual behaviour prior to undergoing circumcision [5, 15]

Given that the teen years and early adulthood are marked as a period of heightened sexual activity and where demand for VMMC is highest (under 18 years) [16], very little is known about the resumption of sexual activity in this age group during the post circumcision healing period. Accordingly, this study investigates: i) the prevalence of the early resumption of sexual activity during the six week healing period amongst sexually active school-going males, and ii) the factors associated with this risk-taking behaviour.

## Methodology

This study, conducted in 2013, took place in the Vulindlela area located in the province of KwaZulu-Natal, South Africa. The HIV prevalence for 12 to 25 year olds in this area is approximately 7% amongst females and 1.4% amongst males [17].

The study participants were HIV negative high school learners who were circumcised at a clinic in Vulindlela operated by The Centre for the AIDS Programme of Research in South Africa’s programme (CAPRISA). CAPRISA performed 5165 circumcisions in a 23 month period, from March 2011 to February 2013, in the Vulindlela area [7]. The 42 schools in Vulindlela targeted by CAPRISA had a total male population of 11 088 learners. The high schools were all mixed sex schools with the number of males in each school ranging from 91 to 892. Male learners who enrolled for circumcision were purposively recruited for this study and interviewed prior to undergoing the procedure and again six weeks post circumcision. The successes and challenges faced with regards to recruitment in the VMMC drive have been well documented elsewhere [7].

The baseline survey purposively sampled 589 young males from which 366 were successfully followed up after six weeks (62% response rate). There was no significant difference in age (p = 0.629) or in sexual activity (p = 0.734) between those that had been lost to follow-up and those that were interviewed. A further 45 respondents were removed from the sample due to inconsistent sexual history data. The realized sample size for this study is 321 male learners. The small sample size can be attributed to the small window period to reach the learner following circumcision. Where the six week healing period ended during school holidays or exam periods, this cohort were inaccessible, whilst high absenteeism rates at the schools further contributed to the attrition of study participants.

The study’s population is school-going males from the ages of 16 to 24 years, with few respondents (< 10%) older than 21. A waiver of Parental/Guardian Informed Consent for learners who were younger than 18 years was sought from the University of KwaZulu-Natal Biomedical Research Ethics Committee. A waiver was sought following the application of the Child Care Act 74 (1983), Section 5.3.1, which states that “*Research involving adolescents who may consent unassisted should be approved only if*:

- The research, including observational research, places the adolescent at no more than minimal risk; and
- The nature of the research is such that, in the opinion of the research ethics committee, the parents or legal guardians or community at large are unlikely to object to the adolescent consenting him or herself to participation in the investigation. The opinion of the research ethics committee must be informed by information gathered from the community concerned and by contributions from the lay members of the committee.

Our preparatory visits to the study site indicated that the parental population was highly mobile, with low literacy rates. Obtaining parental or guardian consent was proving difficult within this setting. It was further argued that this attitudinal or behavioral study brought minimal risk to participants because it assessed learner’s knowledge, attitudes and sexual practices by means of a self-administered questionnaires and only in instances where males did not understand a question were they assisted by fieldworkers. Only males 16 years or over were eligible to participate in the study. In addition to the informed consent documents, we also used a Community Research Support Group (CRSG) to seek community approval for the study. CAPRISA had used the CRSG structure for their on-going programme in the communities where this study was undertaken. We were therefore of the opinion that males over the age of 16 years would be able to understand the implications of participating in the study, that is, completing a self-administered questionnaire on knowledge and attitudes towards VMMC and sexual behavioural practices. Full ethical approval along with the waiver of Parental/Guardian Informed Consent for learners was granted by the University of KwaZulu-Natal Biomedical Research Ethics Committee (REF: BF128/11).

All the interviews were conducted in Zulu at the learner’s respective schools during recess. The fieldworkers were South African men of Zulu ethnicity from the same area, between the ages of 20 and 35 years of age.

The questionnaire had nine focus areas, including: history of STIs, symptoms of TB, sexual history during the previous six weeks, alcohol and drug use during the previous six weeks, condom use in the previous six weeks, sexual behaviour intentions, perceptions of risk of being infected with HIV/AIDS, adverse symptoms following VMMC and an evaluation of the information received during VMMC campaigns.

Two scales were used in this study. The first scale measured intentional risky sexual behaviours. The sexual behaviour intentions scale consisted of three items: “I am going to use a condom the next time I have sex”, “I am going to reduce the number of my sexual partners over the next year” and “I am going to have one sexual partner at a time over the next year”. Response options for these items were “yes”, “no” or “don’t know”. These three items were computed into an index score with a high score indicating greater intentions to practice safe sex in the future. The alpha reliability score for this scale was 0.6.

The second scale focused on the information provided to participants during the VMMC campaign. This scale consisted of three items: “Do you feel you were given enough information about the intervention you participated in”, “Do you feel that the manner in which the information was provided was interesting and useful” and “Do you feel you have enough knowledge about medical circumcision”. Response options for these items were “yes” and “no”. These three items were computed into an index score combined into a count variable with a high score indicating greater satisfaction with the VMMC information provided by councilors at the clinic. The alpha reliability of this scale was 0.6.

For the purposes of analysis, we selected the subsample of males who had ever been sexually active at baseline (n = 138, 43% of the full sample). These individuals were sampled from 25 of the 42 schools in Vulindlela. Sexually active was defined as ever having engaged in anal or vaginal sex. The focus on sexually active males was premised on the fact that they were more likely to have the opportunity to engage in sexual acts during the healing period. In order to address the central study question, the sexually active group was further divided into those who had sex during the healing period (n = 40) and those who did not have sex during the healing period (n = 98).

**Fig 1:**
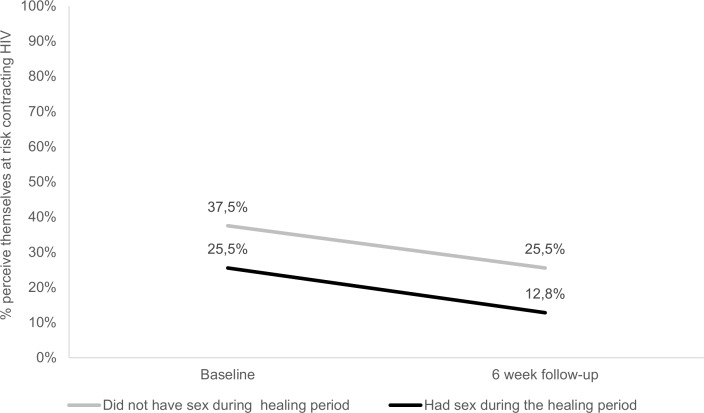
Perceptions of the risk of contracting HIV

**Fig 2:**
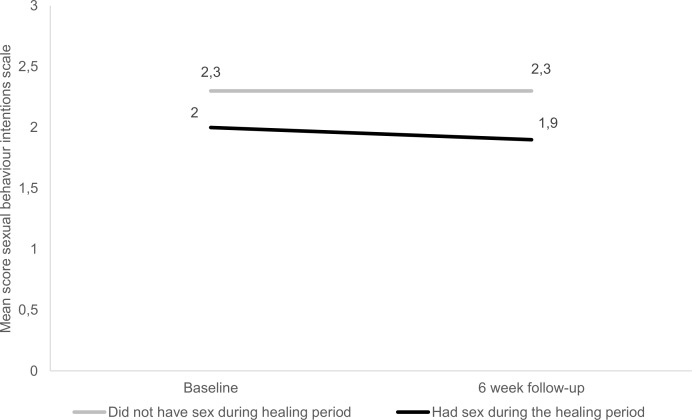
Sexual behaviour intentions of adolescents

Both groups were used as primary comparators for the study analysis and the differences between these groups in terms of socio-demographic characteristics and the two scales were tested with t-tests and chi-square tests. A p-value of less than 0.05 was used as a criterion for a statistically significant difference between the two groups of males, although a p-value of less than 0.10 was noted to highlight relationships between variables that may have been statistically significant if the study had sufficient power. A binomial logistic regression was conducted to assess which factors were significantly associated with whether males would have sex during the healing period. In addition to the logistic regression, a longitudinal analysis was undertaken from the baseline interview to the six week follow-up interview (see results in Figs 1 and 2). This allowed for the identification of possible trends in sexual behaviour intentions and perceived risk of contracting HIV. The analysis of longitudinal data uses generalized linear models to test differences in risk perceptions and sexual behaviour intentions. Data analysis was completed using IBM SPSS version 23 [18].

## Results

One hundred and thirty-eight (43%; 95% CI: 37.5–48.3) of the 321 respondents followed up six weeks post circumcision had been sexually active at baseline. Amongst the 138 sexually active respondents, 29% (n = 40) (95% CI: 21.4–36.6) had anal or vaginal sexual intercourse during the healing period. Only 4% (n = 6) of the sexually active males indicated they masturbated during the six week healing period. Table 1 compares the sample of sexually active learners between those that abstained from sexual activity with those who had engaged in sex during the healing period.

**Table 1:** Descriptive statistics for background, sexual behaviours and VMMC attitudes at six weeks following circumcision

Demographic characteristics	Cohort 1: Reported not being sexually active during the healing period. (n = 98)	Cohort 2: Did have sex during the healing period. (n = 40)	Statistical test
Age (mean, SD)	(17.7, 1.7)	(17.9, 1.8)	t(135) = -.489, p = 0.626
Age			
16–18 yrs. n(%)	49(50)	18(46)	
19–24 yrs. n(%)	49(50)	21(54)	
Grade (Mean, SD)	(9.9, 1.2)	(9.8, 1.0)	t(135) = .557, p = 0.579.
Sexual debut age (Mean, SD)	(15.2, 1.9)	(14.9, 1.9)	*t*(136) = .1.058, p = 0.292,
Number of times alcohol used in healing period (Mean, SD)	(0.2, 1.6)	(0.6, 1.9)	t(136) = -1.085, p = 0.280,
Number of adverse symptoms following MMC (Mean, SD)	(1.9, 1.7)	(2.3, 1.9)	t(136) = -1.119, p = 0.265
MMC information scale (Mean, SD)	(2.7, 0.6)	(2.7, 0.7)	t(136) = 0.034, p = 0.973
Sexual behaviour intentions scale (Mean, SD)	(2.3, 1.0)	(1.9, 1.1)	t(136) = 1.864, p = 0.064.
Number of sex acts last 6 weeks (mean, SD)	n/a	(2.3, 1.6)	
Number of sexual partners last 6 weeks (mean, SD)	n/a	(1.6, 1.2)	
It is important to abstain during 6 week period			χ^2^(2) = .596, p = 0.963
Agree n(%)	75(79)	29(73)	
Uncertain n(%)	10(10)	3(8)	
Disagree n(%)	13(13)	7 (18)	
Important to not engage in risky sex behaviour during healing period			χ^2^(2) = 2.894, p = 0.576
Agree n(%)	77(79)	32(80)	
Uncertain n(%)	6 (6)	0 (0)	
Disagree n(%)	15 (15)	8(20)	
Condoms used in last six weeks			
Consistently n(%)	n/a	12(36)	n/a
Inconsistently n(%)		21(64)	
Have you ever used a condom during sex			χ^2^(1) = 2.500, p = 0.114
Yes n(%)	59(61)	30(75)	
No n(%)	39(38)	10(25)	

Table 1 reveals that there was no statistically significant difference between the two groups in terms of age (p = 0.626) and school grade (p = 0.579). Those that had sex during the healing period are more likely to consume alcohol (0.6 times vs 0.2 times) during the six week healing period. It should be noted that the difference between the two means is not statistically significant (p = 0.280), although the effect size statistic indicates a small to medium difference (Cohen's D = 0.22).

The results show that the 40 males that engaged in sexual activity during the six week healing period participated in two sexual acts and had two sexual partners on average during the healing period. There was no difference at baseline between the two study groups in relation to the number of partners in the previous month (p = 0.563) and number of partners in the previous 6 months (p = 0.701, results not shown here). Just more than three-quarters (75%) of males in the cohort who resumed sexual activity during the healing period indicated having previously used a condom during sexual intercourse, against 61% in the cohort that were sexually active but abstained during the healing period. This difference was not statistically significant (p = 0.114). The males who had sex during the healing period used condoms inconsistently, with just over a third (36%) using a condom during every sex act within the healing period. When comparing consistency of condom usage from the baseline interview to the six week interview (results not shown here), condom usage remained consistent over the study period (42% consistency at baseline to 36% at the 6 week follow-up) for those males engaging in sex during the healing period. While, the decrease in condom usage was not statistically significant (p = 0.189), it is of concern to note the consistent low levels of condom use.

### Multivariate analysis

A binomial logistic regression was run on the cross-sectional data collected at the 6 week interview. The bivariate model focuses on the bivariate relationship between each independent variable and self-reported acts of sex during the healing period. The background model presents the relationship between the learner’s demographic information (age and grade) with self-reported acts of sex during the healing period. The HIV risk behaviour model highlights the relationship between HIV risk behaviours (alcohol use, condom use and age at sexual debut) and self-reported acts of sex during the healing period. The knowledge, attitudes and experiences model focused on the relationship of knowledge, attitudes and experiences of individuals concerning VMMC and HIV (knowledge of abstaining during healing period, satisfaction with information sessions prior to procedure, future sexual behaviour intentions, experience of adverse symptoms following VMMC and self-perceived risk of contracting HIV) with self-reported acts of sex during the healing period. The full model combines all the variables from the background model, HIV risk behaviour model and knowledge, attitudes and experiences model to assess which variable had the strongest association with self-reported acts of sex during the healing period. The full model was statistically significant (see Table 2, p = 0.036), additionally, the Hosmer and Lemeshow test indicated that the model was a good fit for the data (p = 0.526). The independent variables collectively accounted for 31% of the variance in whether males had sex during the healing period. However, one limitation of the final model is that 46 cases were excluded due to missing values in the independent variables.

**Table 2:** Binary logistic regression for the variables associated with engaging in sex during the healing period

Independent variables	Bivariate model		Background model		HIV risk behaviour model		Knowledge, attitudes & experiences model		Full model	
	OR	P	OR	P	OR	P	OR	P	OR	P
Intercept	n/a		0.188	0.464	8.930	0.210	0.897	0.923	39.401	0.390
Age	1.071	0.562	1.138	0.337					1.477	0.059
Grade	0.909	0.576	0.852	0.400					0.848	0.558
Alcohol use in general	1.110	0.317			1.156	0.175			1.178	0.289
Has used condom (No condom used = ref cat)	0.518	0.117			0.428	0.057			0.009	0.014
Age at sexual debut	0.902	0.292			0.875	0.195			0.850	0.297
It is important to abstain during 6 week period	1.062	0.709					0.967	0.893	1.098	0.760
It is important to not engage in risky sex behaviour after 6 weeks healing period	1.029	0.859					0.870	0.646	0.819	0.555
Information provided on MMC	0.990	0.973					0.913	0.783	0.755	0.448
Sexual behaviour intentions	0.720	0.067					0.727	0.151	0.055	0.002
Sexual behaviour intentions*ever used a condom	n/a	n/a					n/a	n/a	6.489	0.010
Adverse symptoms following MMC	1.121	0.265					1.190	0.160	1.115	0.457
Does not perceive self at risk (High risk ref cat)	2.210	0.225					1.760	0.422	1.521	0.603
N	n/a		136		137		95		92	

Notes: Outcome variable is sex during the healing period (0 = abstained during healing period, 1 = had sex during healing period).Full model: R^2^ = 0.214 (Cox & Snell), 0.308 (Nagelkerke). Model *χ*^2^ (12) = 22.175, p = 0.036. Hosmer and Lemeshow test p = 0.526

The bivariate model shows that for every one unit increase in the sexual behaviour intentions of males, the likelihood of having sex during the healing period decreased (OR = 0.720, p = .067). Although the coefficient was nearing statistical significance, these results suggest that those males who had sex during the healing period did not intend to practice safer sex in the future as compared to those males who had abstained. The behaviour model also indicated that having ever used a condom during sex increased the likelihood of having sex during the healing period (OR = 0.428, p = 0.057), although once again it is only nearing statistical significance. There is an interaction between the sexual behaviour intentions scale and whether individuals had ever used a condom (OR = 6.489, p = 0.010). To make sure this result was not based solely on 92 individuals in the final model, this analysis was run with the two original variables (sexual behaviour intentions and ever used a condom) and the interaction term (sexual behaviour intentions by having ever used a condom) in the model which yielded the same results. This result is based on the full sample of 136 cases and not 92 as is the case in the final model in Table 2.

With regards to the interaction term in the regression model, those that had a low score on the sexual behaviour intentions scale (on the intentions to practice safe sex scale) (< 2 out of 3) were more likely to have ever used a condom during sexual intercourse. This finding applies more to the group of males who had sex during the healing period and suggests that previous protective behaviours (having ever used a condom) do not appear to be associated with sexual behavioural intentions. Those males that had sex during the healing period have a far greater incongruence between previous protective behaviours and future intentions.

### Longitudinal analysis of respondents’ beliefs

Fig 1 shows the percentage of males who believed they were at high risk of contracting HIV. There was a 12% reduction for those who had sex during the healing period and a 13% reduction for those that abstained during the healing period between the baseline and six week follow-up interview. The change over time for both groups was not statistically significant (p = 0.159) and the difference between the groups was also not statistically significant (p = 0.149).

At the six week follow-up interview, males that had sex during the healing period were less inclined to adopt future safer sex practices (mean of 2.3 vs 1.9 at 6 week follow-up, p = 0.064) than their counterparts who had abstained during the healing period. Respondents in the cohort that had sex during the healing period were most averse to having only one partner at a time over the next year (results not shown). A quarter of respondents in this cohort indicated a propensity to engage in multiple concurrent relationships. The difference between the two study groups (those who had sex and those who did not have sex during the healing period) was statistically significant as shown in the regression analysis in Table 2. However, the change from baseline to 6 week follow-up for both groups is not statistically significant (see Fig 2; p = 0.466) and we therefore cannot be sure that the mean for both cohorts decreased over this period. The interaction between the group effect (had sex during healing period variable) and time effect (when the interview was conducted either at baseline or six week follow-up) is not statistically significant (p = 0.839). We therefore have to assume that the learner’s sexual behaviour intentions did not diverge over the 6 week period. Overall the data reveals that respondents who did not have sex during the healing period were slightly more likely than respondents who did have sex during the healing period to intend to practice safe sex in the future, as indicated in the statistically significant study group effect.

## Discussion

The purpose of a VMMC programme is to reduce the risk of HIV infection amongst males who have undergone the procedure. However, the early resumption of sexual activity by males during the six week healing period may limit the positive impact of this HIV prevention strategy. Previous results on the early resumption of sexual activity post VMMC are inconsistent. A fairly high percentage (22%) of adult men had sex during the healing period in a South African randomized control trial; although it was shown that the early resumption of sex was not associated with the risk of contracting HIV [14]. By contrast, randomized controlled trials in Uganda (5.4%) and Kenya (3.9%) revealed a lower prevalence of the early resumption of sex following VMMC [3, 19].

It is concerning that 29% of the males reported sexual acts during the healing period. In addition, 64% (n = 21) of these males reported inconsistent condom use during the healing period. Therefore, HIV acquisition amongst this newly circumcised cohort, who used condoms inconsistently during the healing period, remains a real concern. This concern is further amplified by the fact that the HIV prevalence of school-going females in this area was 7% [17]. Furthermore, the prevalence of sex during the healing period in this study is close to the 30% tipping point suggested by Hewett et al [5] which could potentially result in the increase of HIV infections amongst females in the short term. It should be noted that the sample from this study is unlikely to be representative of the 5165 individuals circumcised during the VMMC programme and therefore this conclusion should be interpreted cautiously. More statistical modelling work is therefore needed in the South African context to understand the impact of the early resumption of sex during the healing period on HIV acquisition in the VMMC programme.

The findings pose some important questions regarding the effect of educational activities that precede circumcision in this population. The high numbers of sexually active males who resumed sexual activity in the short period post circumcision suggests that the information received during the VMMC campaigns had a limited effect two respects: delaying sexual activity and promoting consistent condom use. While the general educational content may not have been problematic, as most young men were satisfied with the VMMC information provided, the inability to discourage sexual activity during the healing period needs to be highlighted. Apart from producing messages to target sexual activity during this period, education campaigns should be extended to peers, where research suggests that female sexual partners can influence men to abstain or at least adopt safe sex practices following VMMC [20].

### Limitations

This study is limited by its inability to directly distinguish between those males who made a conscious choice not to have sex during the healing period and those males who did not have an opportunity to have sex during the healing period, but may have done so if presented with an opportunity. Consequently, the comparison between the two cohorts that were sexually active was important, as it is likely that males in both of these groups had similar opportunities to engage in sexual activity; therefore males who have never had sex may not have had the same opportunity to engage in sexual activity as those males included in our analysis. This limitation means that the researchers cannot make assumptions about the agency of the males in choosing to have sex during the healing period.

The study had a fairly low response rate (62%) due to the very small window of opportunity to locate the young males at exactly six weeks. This low response rate affected the sample size. Due to the small sample, the results from these learners cannot be generalised. This type of study warrants a larger sample size for future research. Furthermore, the clustering of males within schools was not taken into consideration as there was not enough statistical power to do this as certain schools only had one or two learners in them who formed part of this study.

A final concern is the self-reported nature of the males’ sexual activity. It is well known that self-reported sexual activity can be unreliable. It has been shown that high frequency sexual behaviours are often reported less consistently than low frequency sexual behaviours because high frequency sexual behaviour respondents are less likely to remember specific instances of sexual activity [21, 22].

## Conclusion

This study has shown that early resumption of sexual activity following VMMC is indeed a concern for policymakers and bodies involved in VMMC promotion. Such an investigation allows for the design of more directed educational campaigns aimed at males who are considering or volunteering for circumcision. The risk of contracting HIV is exacerbated by inconsistent condom use in the group of males that resumed sex during the healing period. The resumption of sexual activity during the healing period places these males at potential risk of contracting HIV.

The study is the first to focus on school-going males undergoing VMMC. It is accepted that young males are entering into a period of sexual exploration which in itself is a period of high risk due to increased sexual activity. VMMC campaigns increasingly focus on school-going male males due to their accessibility [16]. Campaigns must therefore be cognizant of young males who are already engaging in sexual activity to ensure that they are aware of the risks of early sexual resumption post circumcision. The benefits of VMMC decreasing the chances of contracting HIV for males have been extensively researched and verified [3, 4]; however, the engagement in riskier sexual activities by newly circumcised males may reduce the impact of the VMMC programme. Bodies tasked with implementing VMMC programmes must therefore ensure that targeted recipients are well versed in the risk of early sexual resumption post circumcision and should strongly advocate for abstinence during the healing period or the adoption of safe sex practices for these six weeks and beyond.

## References

[1] South African National Strategic Plan on HIV/AIDS, STIs and TB 2012–2016. 2011.

[2] Wawer MJ, Makumbi F, Kigozi G, Serwadda D, Watya S, Nalugoda F, et al Circumcision in HIV-infected men and its effect on HIV transmission to female partners in Rakai, Uganda: a randomised controlled trial. The Lancet. 2007;374(9685):229–37. 10.1016/S0140-6736(09)60998-3 PMC2905212 19616720

[3] Gray RH, Kigozi G, Serwadda D, Makumbi F, Watya S, Nalugoda F, et al Male circumcision for HIV prevention in men in Rakai, Uganda: a randomised trial. The Lancet. 2007;369(9562):657–66. 10.1016/S0140-6736(07)60313-4 17321311

[4] Auvert B, Taljaard D, Lagarde E, Sobngwi-Tambekou J, Sitta R, Puren A. Randomized, Controlled Intervention Trial of Male Circumcision for Reduction of HIV Infection Risk: The ANRS 1265 Trial. PLoS Med. 2005;2(11):e298 10.1371/journal.pmed.0020298 16231970 PMC1262556

[5] Hewett PC, Hallett TB, Mensch BS, Dzekedzeke K, Zimba-Tembo S, Garnett GP, et al Sex with stitches: assessing the resumption of sexual activity during the postcircumcision wound-healing period. AIDS. 2012;26(6):749–56. 10.1097/QAD.0b013e32835097ff 22269970

[6] HIV/AIDS JUNPo. Joint strategic action framework to accelerate the scale-up of voluntary medical male circumcision for HIV prevention in Eastern and Southern Africa. 2011.

[7] Montague C, Ngcobo N, Mahlase G, Frohlich J, Pillay C, Yende-Zuma N, et al Implementation of Adolescent-Friendly Voluntary Medical Male Circumcision Using a School Based Recruitment Program in Rural KwaZulu-Natal, South Africa. PLoS ONE. 2014;9(5):1–7. 10.1371/journal.pone.0096468 PMC4008624 24788339

[8] Eaton LA, Kalichman SC. Risk Compensation in HIV Prevention: Implications for Vaccines, Microbicides, and Other Biomedical HIV Prevention Technologies. Current HIV/AIDS reports. 2007;4(4):165–72. 18366947 10.1007/s11904-007-0024-7 PMC2937204

[9] Ombere SO, Nyambedha EO, Bukachi SA. Wimbo: implications for risk of HIV infection among circumcised fishermen in Western Kenya. Culture, Health & Sexuality. 2015:1–8. 10.1080/13691058.2015.1018949 25774858

[10] Abbott SA, Haberland NA, Mulenga DM, Hewett PC. Female Sex Workers, Male Circumcision and HIV: A Qualitative Study of Their Understanding, Experience, and HIV Risk in Zambia. PLoS ONE. 2013;8(1):e53809 10.1371/journal.pone.0053809 23349745 PMC3547927

[11] Albert LM, Akol A, L'Engle K, Tolley EE, Ramirez CB, Opio A, et al Acceptability of male circumcision for prevention of HIV infection among men and women in Uganda. AIDS Care. 2011;23(12):1578–85. 10.1080/09540121.2011.579939 21732902

[12] Mattson CL, Campbell RT, Bailey RC, Agot K, Ndinya-Achola JO, Moses S. Risk Compensation Is Not Associated with Male Circumcision in Kisumu, Kenya: A Multi-Faceted Assessment of Men Enrolled in a Randomized Controlled Trial. PLoS ONE. 2008;3(6):e2443 10.1371/journal.pone.0002443 18560581 PMC2409966

[13] Westercamp N, Agot K, Jaoko W, Bailey R. Risk Compensation Following Male Circumcision: Results from a Two-Year Prospective Cohort Study of Recently Circumcised and Uncircumcised Men in Nyanza Province, Kenya. AIDS Behav. 2014;18(9):1764–75. 10.1007/s10461-014-0846-4 25047688

[14] Mehta SD, Gray RH, Auvert B, Moses S, Kigozi G, Taljaard D, et al Does Sex in the Early Period After Circumcision Increase HIV-Seroconversion Risk? Pooled Analysis of Adult Male Circumcision Clinical Trials. AIDS (London, England). 2009;23(12):1557–64. 10.1097/QAD.0b013e32832afe95 PMC2772053 19571722

[15] Herman-Roloff A, Bailey RC, Agot K. Factors Associated with the Early Resumption of Sexual Activity Following Medical Male Circumcision in Nyanza Province, Kenya. AIDS Behav. 2012;16(5):1173–81. 10.1007/s10461-011-0073-1 22052231 PMC3677080

[16] Njeuhmeli E, Hatzold K, Gold E, Mahler H, Kripke K, Seifert-Ahanda K, et al Lessons Learned From Scale-Up of Voluntary Medical Male Circumcision Focusing on Adolescents: Benefits, Challenges, and Potential Opportunities for Linkages With Adolescent HIV, Sexual, and Reproductive Health Services. JAIDS Journal of Acquired Immune Deficiency Syndromes. 2014;66:S193–S9. 10.1097/QAI.0000000000000179 24918595

[17] Kharsany AB, Mlotshwa M, Frohlich JA, Zuma NY, Samsunder N, Karim SSA, et al HIV prevalence among high school learners-opportunities for schools-based HIV testing programmes and sexual reproductive health services. BMC public health. 2012;12(1):231. 22439635 10.1186/1471-2458-12-231 PMC3359203

[18] IBM. IBM SPSS Statistics for Windows. 23rd edition Armonk: NY: IBM Corp.

[19] Bailey RC, Moses S, Parker CB, Agot K, Maclean I, Krieger JN, et al Male circumcision for HIV prevention in young men in Kisumu, Kenya: a randomised controlled trial. The Lancet. 369(9562):643–56. 10.1016/S0140-6736(07)60312-2 17321310

[20] Lanham M, L’Engle KL, Loolpapit M, Oguma IO. Women’s Roles in Voluntary Medical Male Circumcision in Nyanza Province, Kenya. PLoS ONE. 2012;7(9):e44825 10.1371/journal.pone.0044825 23028634 PMC3446991

[21] Fenton KA, Johnson AM, McManus S, Erens B. Measuring sexual behaviour: methodological challenges in survey research. Sexually Transmitted Infections. 2001;77(2):84–92. 10.1136/sti.77.2.84 11287683 PMC1744273

[22] Brener ND, Billy JOG, Grady WR. Assessment of factors affecting the validity of self-reported health-risk behavior among adolescents: evidence from the scientific literature. Journal of Adolescent Health. 2003;33(6):436–57. 14642706 10.1016/s1054-139x(03)00052-1

